# Absence of the MGMT protein as well as methylation of the *MGMT* promoter predict the sensitivity for temozolomide

**DOI:** 10.1038/sj.bjc.6605712

**Published:** 2010-06-01

**Authors:** K A van Nifterik, J van den Berg, W F van der Meide, N Ameziane, L E Wedekind, R D M Steenbergen, S Leenstra, M V M Lafleur, B J Slotman, L J A Stalpers, P Sminia

**Affiliations:** 1 Department of Radiation Oncology, VU University Medical Center, P.O Box 7057, Amsterdam 1007 MB, The Netherlands; 2 Department of Pathology, VU University Medical Center, P.O Box 7057, Amsterdam 1007 MB, The Netherlands; 3 Department of Clinical Genetics, VU University Medical Center, P.O Box 7057, Amsterdam 1007 MB, The Netherlands; 4 Department of Neurosurgery, St Elisabeth Ziekenhuis, Tilburg, The Netherlands; 5 Department of Radiotherapy, Academic Medical Center, Amsterdam, The Netherlands

**Keywords:** MGMT, temozolomide, glioma, prediction

## Abstract

**Background:**

The DNA repair protein O^6^-methylguanine-DNA methyltransferase (MGMT) can cause resistance to the alkylating drug temozolomide (TMZ). The purpose of this study was to determine the relationship between the MGMT status, determined by means of several techniques and methods, and the cytotoxic response to TMZ in 11 glioblastoma multiforme (GBM) cell lines and 5 human tumour cell lines of other origins.

**Methods:**

Cell survival was analysed by clonogenic assay. The MGMT protein levels were assessed by western blot analysis. The *MGMT* promoter methylation levels were determined using methylation-specific multiplex ligation-dependent probe amplification (MS-MLPA) and quantitative real-time methylation-specific PCR (qMSP). On the basis of the results of these techniques, six GBM cell lines were selected and subjected to bisulphite sequencing.

**Results:**

The MGMT protein was detected in all TMZ-resistant cell lines, whereas no MGMT protein could be detected in cell lines that were TMZ sensitive. The MS-MLPA results were able to predict TMZ sensitivity in 9 out of 16 cell lines (56%). The qMSP results matched well with TMZ sensitivity in 11 out of 12 (92%) glioma cell lines. In addition, methylation as detected by bisulphite sequencing seemed to be predictive of TMZ sensitivity in all six cell lines analysed (100%).

**Conclusion:**

The MGMT protein expression more than *MGMT* promoter methylation status predicts the response to TMZ in human tumour cell lines.

Glioblastoma multiforme (GBM) is the most frequent and most malignant primary brain tumour. Despite surgery, adjuvant radiotherapy and chemotherapy, GBM often recur. Addition of temozolomide (TMZ) to fractionated radiotherapy for newly diagnosed GBM improved the median survival from 12 to 14 months and the 5-year overall survival from 1.9 to 9.8% (Stupp *et al*, 2005, 2009).

TMZ, an alkylating cytostatic drug, acts through methylation of the O^6^ position of guanine; the resulting DNA lesions are considered to be responsible for the cytotoxic effect (Stevens *et al*, 1987; Tisdale, 1987). Presence of the DNA repair protein O^6^-methylguanine-DNA methyltransferase (MGMT) can cause resistance to alkylating agents, including TMZ, by removing the methyl group from the O^6^ position of guanine (Brennand and Margison, 1986).

Both transcriptional silencing of the *MGMT* gene by methylation of the CpG dinucleotides (CpGs) in the promoter region as well as absence of MGMT protein have been associated with a good clinical response to alkylating agents in general and in particular to TMZ in patients with an anaplastic astrocytoma or GBM (Esteller *et al*, 2000; Hegi *et al*, 2005; Chinot *et al*, 2007; Nagane *et al*, 2007; Sadones *et al*, 2009). However, the few studies comparing different approaches to determination of the MGMT status all show a lack of correlation between MGMT protein expression and *MGMT* promoter methylation (Brell *et al*, 2005; Jeuken *et al*, 2007; Preusser *et al*, 2008; Rodriguez *et al*, 2008; Yachi *et al*, 2008).

Prediction of the TMZ sensitivity in clinical tumour samples would greatly improve the patients’ treatment. However, to have clinical value, such an assay needs to be accurate, reliable and relatively convenient.

The purpose of this study was to determine whether the *in vitro* cytotoxic response to TMZ, evaluated by clonogenic assay, is associated with either MGMT expression and/or promoter methylation. In a panel of 16 human cancer cell lines, mostly derived from human GBM, MGMT protein expression was measured by western blot analysis. The *MGMT* promoter methylation levels were assayed by methylation-specific multiplex ligation-dependent probe amplification (MS-MLPA), methylation-specific PCR (MSP), quantitative real-time MSP (qMSP) and bisulphite sequencing.

## Materials and methods

### Cell lines

Sixteen cell lines were cultured at 37°C in medium containing foetal calf serum (FCS), 2 mmol l^–1^ L-glutamine, 100 IU ml^–1^ penicillin and 100 IU ml^–1^ streptomycin (all from Invitrogen, Groningen, The Netherlands). The PC-3 cell line (prostate adenocarcinoma) was cultured in RPMI-1640 medium with 10% FCS. A-431 (epidermoid carcinoma), Gli-6 (GBM; Fehlauer *et al*, 2000) and HT-29 (colorectal adenocarcinoma) were cultured in Dulbecco's modified Eagle's medium (D-MEM) with 10% FCS. In D-MEM with 20% FCS, AMC 3046, AMC 3344, VU-28, VU-98, VU-109, VU-110 and VU-122 were cultured (all GBM; van Nifterik *et al*, 2007). The above-mentioned 11 cell lines were cultured in a 7% CO_2_-humidified atmosphere. Furthermore, five cell lines were cultured in a humidified atmosphere without CO_2_ using Leibovitch L15 medium with 10% FCS: D384 (astrocytoma grade III; Balmforth *et al*, 1986), Hs 683 (GBM), SW 1573 (alveolar cell carcinoma), T98G (GBM) and U251 (GBM). Experiments were performed on cells from the same batch with the same cell passage number, except for western blot analysis of the T98 cell line.

### Clonogenic cell survival after TMZ treatment

The experiment was performed as described earlier (van Nifterik *et al*, 2007). After 24 h exposure of TMZ, the medium was replaced either with fresh conditioned medium (AMC 3046, VU-109 and VU-110) or fresh normal medium (all other cell lines) for the remaining part of the clonogenic assay. Average plating efficiencies (for the untreated cell lines) were 50% (A-431), 17% (AMC 3046), 12% (AMC 3344), 62% (D384), 49% (Gli-6), 30% (HS683), 67% (HT-29), 40% (PC-3), 56% (SW1573), 42% (T98), 33% (U251), 4% (VU-28), 7% (VU-98), 16% (VU-109), 6% (VU-110) and 36% (VU-122). The results for AMC 3046, AMC 3344, VU-28, VU-98, VU-109, VU-110 and VU-122 have already been published (van Nifterik *et al*, 2007).

### MGMT protein expression by western blot analysis

The analysis was performed as described earlier (van Nifterik *et al*, 2007) except that for the cell line HT-29, because of an abundance of targeted protein, 25 *μ*g instead of 50 *μ*g of protein sample were used. For western blot analysis of AMC 3344 also 100 *μ*g of protein were used. Colon carcinomas, in this study HT-29, are a positive control for the MGMT protein. Membranes were checked for successful protein transfer and loading control by incubation of the membranes for 1 min in ponceau S.

### Methylation analysis

#### *MGMT* promoter methylation analysis by MS-MLPA

**Figure 1:**
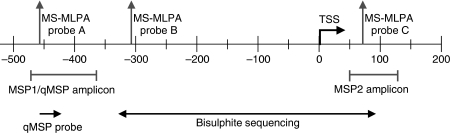
Locations on the *MGMT* promoter for the amplicons of the primer sets for MSP1/qMSP, MSP2, and bisulphite sequencing, and locations for probe qMSP and MS-MLPA probes A, B and C. TSS=transcription start site.

The MS-MLPA is a semi-quantitative method for methylation profiling using a methylation-sensitive restriction enzyme (Nygren *et al*, 2005). The SALSA MS-MLPA kit ME011 (MRC-Holland, Amsterdam, The Netherlands) was used to determine the promoter methylation status of the *MGMT* gene. The kit contains three different probes that specifically target three CpGs within the *MGMT* promoter region (Figure 1): MS-MLPA A, MS-MLPA B and MS-MLPA C, located from −459 to −458, −313 to −312 and 72 to 73 base pairs (bp) from the transcription start site (TSS), respectively. The MS-MLPA was performed with approximately 100 ng DNA according to the protocol provided by the manufacturer. The amplified PCR products were separated by electrophoresis on an ABI PRISM 3730 fragment analyser (Applied Biosystems, Foster City, CA, USA) and analysed using Genemarker analysis software version 1.5 (SoftGenetics, LLC, State College, PA, USA). Data are presented in percentage of methylation (mean of two independent experiments) and classified as low (0–40%), intermediate (40–75%) and high (75–100%).

#### *MGMT* promoter methylation analysis by MSP

Methylation-specific PCR is based on the amplification of bisulphite-converted DNA, which can distinguish specifically between methylated and unmethylated DNA with a high sensitivity (Herman *et al*, 1996). Genomic DNA isolated from cell lines was subjected to bisulphite modification using the EZ DNA Methylation kit (Zymo Research, Orange, CA, USA) according to the protocol provided by the manufacturer. Sodium bisulphite converts unmethylated cytosines to uracils, whereas methylated cytosines are unaffected. Two MSPs were performed targeting two CpG-rich regions within the *MGMT* promoter using primer sets listed in Table 1. The forward primers of MSP1 (3 CpGs) and MSP 2 (5 CpGs; Esteller *et al*, 1999) each contain a CpG that is also detectable by MS-MLPA probes A and C, respectively (Figure 1). The housekeeping gene *β*-actin (ACTB) was used as internal control of both bisulphite conversion DNA quality (Harden *et al*, 2003). In the reaction, 50 ng bisulphite-treated genomic DNA was amplified with FastStart Taq PCR buffer (Roche Diagnostics, Almere, The Netherlands) with 1.5 mM MgCl_2_, 0.5 *μ*M primers, each dNTP at 200 *μ*M, and 1.25 U of FastStart Taq DNApolymerase in a total volume of 25 *μ*l. Amplification reaction was carried out in a GeneAmp PCR system 9700 using the following conditions: 95°C for 4 min, followed by 38 (MSP1 and MSP2) or 40 (ACTB) cycles of 95°C for 30 s, 61°C (MSP1) or 59°C (MSP2) or 60°C (ACTB) for 30 s and 72°C for 45 s, with a final extension of 72°C for 4 min. The cervical cancer cell line CaSki (which is methylated for the *MGMT* gene) was used as positive control, whereas unmethylated (primary keratinocytes), unmodified DNA and H_2_O were included as negative controls. The PCR products were detected by UV light on a 2% agarose gel stained with ethidium bromide. A 100-bp DNA ladder (Amersham Biosciences, Buckinghamshire, UK) was used as a marker. All MSP reactions were performed in duplicate.

#### *MGMT* promoter methylation analysis by qMSP

The qMSP is based on the same principle as MSP, but uses a probe, in this case a Taqman probe, for real-time quantification of amplification products (Vlassenbroeck *et al*, 2008). The primers and probe included in the qMSP are listed in Table 1 (see also Figure 1); the same primer set of MSP2 (Esteller *et al*, 1999) was used for the quantitative format. For the amplification reaction, 2.5 *μ*l bisulphite-treated DNA was added to 9.5 *μ*l amplification mix containing 1 × Quantitect Probe mix (Qiagen, Venlo, The Netherlands), 0.8 *μ*M of each primer and 0.208 *μ*M of the 5′JOE/3′BHQ1-labelled probe. Amplification and real-time measurement were performed in the 7500Fast ABI system under the following conditions: 15 min at 95°C followed by 45 cycles of 30 s at 95°C and 1 min at 60°C. The reaction included multiple water blanks, as negative control, and serial dilutions of the CaSki cell line. As reference, the gene *ACTB* was used to control bisulphite conversion and total input DNA (Harden *et al*, 2003). Promoter methylation levels were calculated with the formula [(target gene/*ACTB*) × 1000]. The cutoff value for discrimination between methylation levels was >200=methylated, and <200=unmethylated or low methylated. All samples were tested in duplicate. When discrepant results were obtained, the samples were analysed in quadruplicate and scored positive if at least two reactions were positive.

#### *MGMT* promoter methylation analysis by bisulphite sequencing

Bisulphite sequencing was performed in an area of the *MGMT* promoter not tested before with MS-MLPA, MSP or qMSP (Figure 1). For bisulphite-sequencing analysis, bisulphite-treated genomic DNA of the GBM cell lines AMC 3344, VU-28, VU110, Hs 683, U251 and the astrocytoma grade III cell line D384 (selected on the basis of the results of western blot and MS-MLPA) was amplified using primers listed in Table 1. The PCR mixtures contained 50 ng bisulphite-treated genomic DNA, FastStart Taq PCR buffer (Roche Diagnostics) with 1.5 mM MgCl_2_, 0.5 *μ*M primers, each dNTP at 200 *μ*M, and 1.25 U of FastStart Taq DNApolymerase in a total volume of 25 *μ*l. Amplification reaction was carried out in a GeneAmp PCR system 9700 using the following conditions: 95°C for 4 min, followed by 40 cycles of 95°C for 30 s, 55°C for 30 s and 72°C for 45 s, with a final extension of 72°C for 4 min. Amplified PCR products were purified using the ExoI and SAP (USB Europe GmbH, Staufen, Germany) according to the manufacturer's specifications, and were sequenced directly using the forward or the reverse primer and the BigDye Terminator v3.1 Cycle Sequencing kit (Applied Biosystems). After each sample was purified with the BigDye XTerminator Purification kit, each sequencing product was subsequently analysed by electrophoresis in the 3130 Genetic Analyser (Applied Biosystems). Sixty-two CpGs were analysed, that is every CpG located between −329 and +91 bp from the TSS.

## Results

### Clonogenic cell survival after TMZ treatment

**Figure 2:**
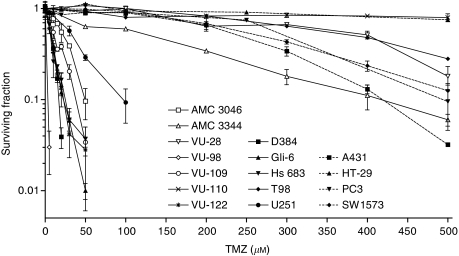
Clonogenic cell survival after 24 h exposure of 16 cancer cell lines to temozolomide (TMZ). The results for AMC 3046, AMC 3344, VU-28, VU-98, VU-109, VU-110 and VU-122 have already been published (van Nifterik *et al*, 2007). Error bars represent the mean±s.d. (*n*=2).

A panel of 16 human tumour cell lines, including 12 gliomas (11 GBM and 1 astrocytoma grade III) and 4 other carcinoma cell lines, was tested for their response to 24 h treatment with different concentrations of TMZ. Cell survival data presented in Figure 2 show that two distinct groups of cell lines can be distinguished: TMZ sensitive and TMZ resistant. The two separate groups can also be shown by ascertaining the surviving fraction (SF) of the cell lines after exposure to 250 *μ*M TMZ, as depicted in Table 2. Cell lines that did not survive this treatment (SF=0) were defined as sensitive for TMZ, whereas cell lines showing at least an SF of 0.4 were considered TMZ resistant.

### MGMT protein expression

**Figure 3:**
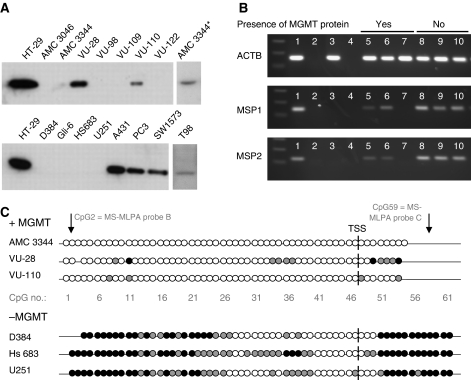
(**A**) Western blot analysis for the expression of O^6^-methylguanine-DNA methyltransferase (MGMT) on a panel of four carcinoma (A-431, HT-29, PC-3 and SW1573) and 12 glioma cell lines. For all samples, 50 *μ*g of total protein were tested, except for the cell line HT-29 (25 *μ*g). The AMC 3344^*^ was also analysed with 100 *μ*g. (**B**) Methylation-specific PCR (MSP) results for the *MGMT* gene promoter using the primer sets MSP1, MSP2 en control (ACTB) for a selection of six cell lines. 1: CaSki (cervical cancer cell line/positive control), 2: unmodified DNA (negative control), 3: unmethylated DNA (primary keratinocytes/negative control), 4: H_2_O, 5: AMC 3344, 6: VU-28, 7: VU-110, 8: D384, 9: HS683, 10: U251. (**C**) Summary of bisulphite sequencing in six GBM cell lines (three with MGMT expression and three without). A total of 62 CpG dinucleotides (CpGs) within the promoter region of *MGMT* were analysed and are represented as circles. Each row represents one individual cell line. Closed circles represent cytosine (methylated), open circles represent uracil (unmethylated) and grey circles represent a peak with both cytosine and uracil.

Sixteen cell lines were tested for expression of the MGMT protein by western blot analysis to investigate its ability to predict cell survival after TMZ exposure. As shown in Figure 3A, eight cell lines exhibit expression of the MGMT protein, including four GBM (AMC 3344, VU-28, VU-110 and T98) and four other carcinomas (A-431, HT-29, PC-3 and SW1573). The levels of expression ranged from high (HT-29) to (very) low (AMC 3344). The other eight cell lines (7 GBM and 1 astrocytoma grade III) did not express detectable levels of the MGMT protein. The MGMT protein was detected in all TMZ-resistant cell lines, whereas no MGMT protein could be detected in cell lines that were TMZ sensitive.

### Methylation analysis

To determine the methylation status of the promoter of the *MGMT* gene, several techniques were used: MS-MLPA (semi-quantitative), MSP, qMSP (quantitative) and bisulphite sequencing.

#### *MGMT* promoter methylation analysis by MS-MLPA

The promoter methylation levels in all 16 cell lines as determined by MS-MLPA were obtained using three probes (directed against different CpGs in the *MGMT* promoter). The mean results of two independent experiments are shown in Table 2. Most regions with low methylation levels (0–40%) were found in the group of cell lines that were resistant to TMZ. However, low methylation levels were also found for probe B in the TMZ-sensitive cell lines VU-98 and VU-122. In contrast, high methylation levels (75–100%) were found mainly in the group of cell lines that were sensitive for TMZ. However, high methylation levels were also found for probe A in the TMZ-resistant cell lines HT-29, SW 1573 and T98. The areas with intermediate methylation levels (40–75%) appeared in both TMZ sensitive and resistant cell lines. A limited association between TMZ sensitivity and *MGMT* promoter methylation was found (Table 2). Presence of at least 2 out of 3 low (0–40%) methylated CpGs determined resistance to TMZ in 5 out of 8 cell lines (63%). Cell lines sensitive to TMZ could only be predicted when at least 2 out of 3 CpGs were highly (75–100%) methylated. This was the case in 4 out of 8 cell lines (50%). For none of the in-between results (i.e. all other combinations of low, intermediate, and high methylated CpGs) was a match found with sensitivity for TMZ in 7 out of 16 cell lines (44%). Overall, MS-MLPA was predictive for TMZ sensitivity in 9 out of 16 cell lines (56%).

#### *MGMT* promoter methylation analysis by MSP

Two different primer sets for different locations within the *MGMT* promoter were used to determine the methylation status by MSP. The MSP1 covers the MS-MLPA probe A and MSP2 covers MS-MLPA probe C (see Figure 1). The results for the primer sets MSP1 and MSP2 (Table 2; Figure 3B) showed methylation positivity for all but two cell lines. Although most cell lines tested positive, there was a clear difference in intensity of PCR products detected. Apparently, the MSP assays are very sensitive, thus necessitating a quantitative assay to allow a better discrimination between the levels of MGMT methylation.

#### MGMT promoter methylation analysis by qMSP

The qMSP probe was designed for the MSP1 region to gain insight into the relative methylation levels in this region of the promoter of the *MGMT* gene. The methylation levels as determined by qMSP are presented as ratios, which indicate the relative amount of methylated *MGMT* (as compared with *ACTB*). The results are shown in Table 2. The ratios for the TMZ-resistant group range from 10.49 to 1338.38, whereas the ratios for the TMZ-sensitive group range from 244.83 to 1460.92. All eight cell lines sensitive to TMZ showed methylation (>200). The four GBM cell lines that were resistant to TMZ showed no or low methylation levels (<200), except for cell line T98. For the four non-glioma cell lines, the opposite was found, these cell lines showed high levels of methylation, except for cell line PC-3 (1 out of 4 cell lines (25%)). Therefore, no association was found between the qMSP data and the cytotoxic response of the non-glioma cell lines to 250 *μ*M TMZ. The TMZ-resistant GBM cell lines (with the exception of T98) and the TMZ-sensitive glioma cell lines showed a good relationship between qMSP data and the cytotoxic response to TMZ (11 out of 12 cell lines (92%)). The qMSP data also showed a good association with data from MS-MLPA probe A.

#### *MGMT* promoter methylation analysis by bisulphite sequencing

On the basis of the results of earlier analyses (Table 2), six GBM cell lines (three with and three without expression of the MGMT protein) were analysed by bisulphite sequencing (in total 62 CpGs; see Figure 1). A summary of the bisulphite sequence results is shown in Figure 3C. All 62 CpGs were completely unmethylated in cell line AMC 3344 and almost completely unmethylated in cell lines VU-28 and VU-110. Unfortunately, we were not able to interpret the results at the 3′ end of the PCR product in these cell lines. In the MGMT-negative cell lines D384, Hs 683 and U251, the 25 CpGs (2–26) located at the 5′ end (−313 → −153 bp from TSS) were completely methylated or contained both methylated and unmethylated CpGs. In addition, the 12 CpGs (51–62) located at the 3′ end were completely or almost completely methylated (+29 → +90 bp from TSS). Methylation levels in these two CpG locations seem to correlate well with TMZ sensitivity. No or partial methylation was detected at CpGs 27–50. The 5′ and 3′ region cover the MS-MLPA probes B (CpG 2) and C (CpG 59) and these bisulphite-sequencing results show an excellent relationship with data from MS-MLPA probe B and MS-MLPA probe C. Bisulphite sequencing predicted TMZ sensitivity in all six tested cell lines (100%).

## Discussion

In this study, we determined the sensitivity to TMZ, assessed by clonogenic cell survival, in a panel of human tumour cell lines, mostly derived from GBM. Sensitivity of TMZ correlated with the *MGMT* promoter methylation status (assessed by MS-MLPA, MSP, qMSP and bisulphite sequencing) and MGMT protein expression (western blot).

**Figure 4:**
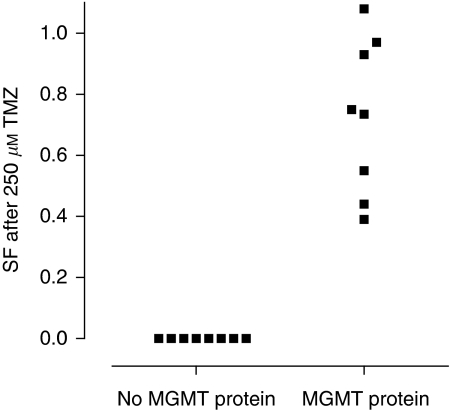
The relationship between temozolomide (TMZ) sensitivity (as indicated by the surviving fraction (SF) after 24 h of 250 *μ*M TMZ) and O^6^-methylguanine-DNA methyltransferase (MGMT) protein expression as determined by western blot analysis.

Methylation of the CpGs in the promoter region of the *MGMT* gene can cause transcriptional silencing, resulting in an absent gene product (i.e. the MGMT protein), which in turn is responsible for failure of repair of the (TMZ-induced) O^6^-methylated guanine lesions in the DNA. Transcriptional silencing of the *MGMT* gene by promoter methylation has been shown in cell lines as well as in human tumour tissues (Qian and Brent, 1997; Watts *et al*, 1997; Esteller *et al*, 1999). The fact that in this study, the absence of the protein indeed reflected cells that are sensitive to TMZ, as shown in Figure 4, agrees with these earlier findings. It is worth noting that expression of the MGMT protein showed a 100% positive match with TMZ resistance, with undetectable levels of MGMT protein in TMZ-sensitive cells and varying levels of MGMT protein in TMZ-resistant cells.

Similarly, Hermisson *et al* (2006) showed that MGMT activity correlated with MGMT expression and clonogenic survival after TMZ exposure in human glioma cells. Although Bobola *et al* (1996) showed that MGMT is a determinant of resistance in medulloblastoma and glioma-derived cell lines, they found disparity in the contribution of MGMT to TMZ sensitivity. They state that the lack of correlation between the amount of MGMT expression and the degree of sensitivity to TMZ, and the variability in cytotoxicity in cell lines without MGMT indicate that other factors are involved in resistance. This could also be the case in the cell lines tested in our study, as we found disparities that were similar to those of Bobola *et al* (1996). For instance, the mismatch repair system or p53 could also be involved in resistance to TMZ in glioma cells.

This study shows that the detection of DNA methylation as a predictor of TMZ response is highly dependent on the technique used and the CpGs analysed. The probes of MS-MLPA had limited predictability (56%). Using MSP in a conventional manner seemed too sensitive, whereas conversion to a quantitative assay (qMSP) enabled a better prediction for glioma cells (92%) based on levels of methylation. Bisulphite-sequencing analysis predicted TMZ sensitivity in all tested samples (100%) and confirmed that MGMT methylation is heterogeneous, as even in the TMZ-resistant cell lines, there are regions in the *MGMT* promoter that remain unmethylated, that is from CpGs 27 to 50. Consequently, these regions should be avoided when designing methylation assays for clinical applications. Furthermore, bisulphite sequencing confirmed qMSP and part of the MS-MLPA results and showed two important regions in the promoter of the *MGMT* gene that are of interest for the relationship between *MGMT* promoter methylation and silencing of the *MGMT* gene. First, the regions in the promoter indicated by the primers and probe of the qMSP technique (−392 → −368 bp from TSS) extended with the location of CpGs 1 to 26 as analysed by bisulphite sequencing (−328 → −153 bp from TSS), including the CpGs from MS-MLPA probes A and B. The second area is located upstream from the TSS covered by CpGs 51 to 62 as analysed by bisulphite sequencing and by the MSP2 primers, also including the CpG from MS-MLPA probe C (+29 → +131 bp from TSS). This information can be used to design new primers and/or probes for MS-MLPA and/or qMSP.

In the literature, a limited amount of CpGs is usually tested for determination of the methylation status. The MSP primer set used in this study (MSP2) has been used in other studies (Esteller *et al*, 1999, 2000; Brell *et al*, 2005; Jeuken *et al*, 2007; Rodriguez *et al*, 2008; Yachi *et al*, 2008). Primer sets that are also often used are located close to or in the same area as MSP2 (Hegi *et al*, 2005; Vlassenbroeck *et al*, 2008; Dunn *et al*, 2009; Sadones *et al*, 2009). Although this seems to be a good location for analysis of methylation patterns, perhaps other and/or more combinations of methylated CpGs would be better predictors of blocking transcription of the *MGMT* gene, hence silencing the gene, which may predict TMZ sensitivity. For instance, the area farther downstream of the TSS (−392 → −153 bp from TSS) may also prove to be of interest.

Studies with human glioma tumour samples often revealed a lack of correlation between MGMT protein expression and *MGMT* promoter methylation status (Brell *et al*, 2005; Jeuken *et al*, 2007; Preusser *et al*, 2008; Rodriguez *et al*, 2008; Yachi *et al*, 2008). Nevertheless, patient studies that investigated the relationship between either MGMT protein expression or *MGMT* promoter methylation and survival of patients with a GBM who had received TMZ as adjuvant chemotherapy have shown promising results. For instance, two studies showed a (negative) correlation between MGMT protein expression and patient survival (Chinot *et al*, 2007; Nagane *et al*, 2007). Other studies found that *MGMT* promoter methylation correlated with patient survival (Hegi *et al*, 2005; Dunn *et al*, 2009; Sadones *et al*, 2009).

Often no correlation is found between promoter methylation and protein expression and both can have prognostic significance in relation to survival of patients treated with TMZ for GBM. Possible explanations may be found in imperfections of the experiment design and/or techniques used. In case of promoter methylation analysis, the promoter region chosen to be investigated may be of influence on the outcome, as described before. In this respect, the technique of MS-MLPA is, despite all its advantages, limited as it can only determine the methylation status of CpGs that are situated within an HhaI restriction site. Furthermore, MSP analysis results may fail in a significant amount of the samples (Hegi *et al*, 2005; Rodriguez *et al*, 2008). Another aspect of many techniques is that differences in MGMT status between individual cells cannot be detected, even though several studies have shown the intratumoural heterogeneity of human gliomas for MGMT protein expression (Citron *et al*, 1991; Belanich *et al*, 1996). Moreover, discrimination between tumour and non-tumour cells in clinical tumour samples is not possible with many of the techniques used and the outcome may be distorted by contamination with non-tumour cells. Techniques such as MSP, qMSP, bisulphite sequencing and WB cannot discriminate. *In vitro* studies, as the present one, do not have this complication, as tumour cell lines are pure neoplastic cell populations. In this respect, immunohistochemistry, determining the expression of the MGMT protein per individual cell, may be a better technique for screening human tumour samples. Nevertheless, immunohistochemistry also has disadvantages, as reported in several studies (Jeuken *et al*, 2007; Capper *et al*, 2008; Preusser *et al*, 2008; Rodriguez *et al*, 2008). Among others, problems were reported with interobserver variability and interpretation of MGMT expressing non-neoplastic infiltrating cells. In addition, suitable techniques such as bisulphite sequencing are often too complex, time consuming and expensive for routine application on tumour samples. On the other hand, techniques such as MSP or immunohistochemistry are easy-to-use and cost-efficient, but have other disadvantages.

In conclusion, MGMT protein expression more than the level of *MGMT* promoter methylation predicts the response to TMZ in human tumour cell lines. Future research should focus on gaining more knowledge of methylation patterns in the promoter region and their relationship with protein expression. In this respect, choosing the appropriate assay that will be accurate, reliable and convenient enough will be of great importance for application in the clinical setting.

## Figures and Tables

**Table 1:** Primer and probe sequences, location and amplicon of *MGMT* gene promoter for the methylation-specific PCR (MSP), quantitative real-time MSP (qMSP) and primer sequences of *MGMT* promoter for bisulphite-sequencing technique

**Name**	**Sequence (5′−3′)**	**… bp from TSS**	**Amplicon (bp)**
MSP1+qMSP MGMT primers	Fw: GATTTTTATTAAGCGGGCGTC	−476 → −456	109
	Rv: CTTTTCCTATCACAAAAATAATCCG	−392 → −368	
			
qMSP MGMT probe	JOE TCCTAAAAACGCGCGAAAATCGTAAAA BHQ1	−453 → −428	
			
MSP2 MGMT primers (Esteller *et al*, 1999)	Fw: TTTCGACGTTCGTAGGTTTTCGC	51 → 73	81
	Rv: GCACTCTTCCGAAAACGAAACG	110 → 131	
			
Bisulphite-sequence primers	Fw: GGTAAATTAAGGTATAGAGTTTTAGG	−354 → −329	464
	Rv: ACCCAAACACTCACCAAAT	91 → 109	

Abbreviations: TSS=transcription start site; bp=base pairs; Fw=forward primer; Rv=reverse primer.

**Table 2:**
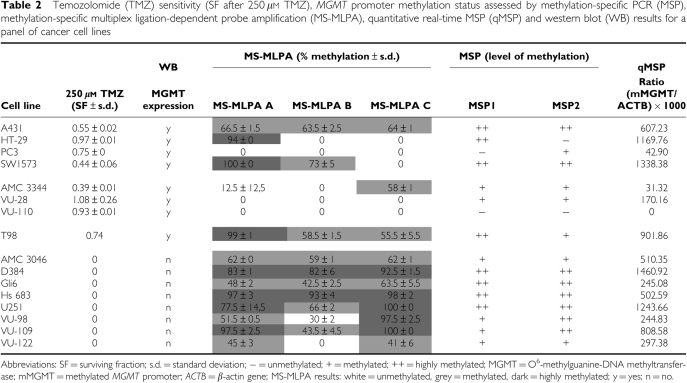
Temozolomide (TMZ) sensitivity (SF after 250 *μ*M TMZ), *MGMT* promoter methylation status assessed by methylation-specific PCR (MSP), methylation-specific multiplex ligation-dependent probe amplification (MS-MLPA), quantitative real-time MSP (qMSP) and western blot (WB) results for a panel of cancer cell lines
